# Psychopathologies and socioeconomic status as risk indicators for periodontitis: a survey-based investigation in German dental practices

**DOI:** 10.1007/s00784-021-04263-2

**Published:** 2021-11-08

**Authors:** Maria Lenk, Barbara Noack, Kerstin Weidner, Katrin Lorenz

**Affiliations:** 1 Department of Psychotherapy and Psychosomatics, Carl Gustav Carus Faculty of Medicine, Technische Universität Dresden, Fetscherstr. 74, 01307 Dresden, Germany; 2 Department of Periodontology, Technische Universität Dresden, Fetscherstr, 74, 01307 Dresden, Germany

**Keywords:** Periodontitis, Dental anxiety, Socioeconomic status, Mental disorders

## Abstract

**Objectives:**

Periodontitis is a highly prevalent multifactorial disease associated with various mental disorders. However, study results about this association are still contradictory. One methodological reason could be the neglect of potential confounders, such as socioeconomic factors or mental comorbidity. Our study examined a wide range of potential psychosocial risk indicators to identify those with relevant associations to periodontitis.

**Materials and methods:**

In a cross-sectional study, 111 patients with periodontitis (PERIO) (> 30% teeth with approximal attachment loss ≥ 5 mm) and 110 patients without periodontitis (NON-PERIO) were recruited in four dental practices in Germany. Clinical attachment loss, pocket depth, plaque, bleeding on probing, and DMFT were measured. Psychopathologic symptoms and socioeconomic status were recorded using self-report questionnaires (DAS, PHQ-8, GAD-7, CTS, SCOFF, AUDIT, FTND, SSS-8, SES).

**Results:**

The PERIO group reported significantly lower socioeconomic status (Cohen’s *d* = 0.49) and higher psychopathological symptom burden than the NON-PERIO regarding dental anxiety (*d* = 0.86) and avoidance behavior, nicotine dependency (*d* = 0.84), depressiveness (*d* = 0.46), general anxiety (*d* = 0.45), somatic symptoms (*d* = 0.42), and childhood traumatization (*d* = 0.34). No significant group differences existed for alcohol abuse and eating disorders. Dental anxiety was the strongest predictor of periodontitis and showed significant correlations with other psychopathologies and social status.

**Conclusions:**

Out of all psychosocial factors, socioeconomic status and dental anxiety showed the greatest association with periodontitis.

**Clinical relevance:**

Dentists should encourage socially disadvantaged and dentally anxious patients in the utilization of prevention and dental care. Furthermore, physicians and psychotherapists can contribute to the early detection of dental anxiety, oral diseases, and avoidance behavior.

## Introduction

Periodontitis is a highly prevalent multifactorial inflammatory disease associated with dysbiotic plaque biofilm, as recently stated at the World Workshop in 2017 [1]. According to the concept of pathogenesis by Page and Kornman [2], acquired factors like smoking, uncontrolled diabetes, and genetic disposition can modify the immune-inflammatory response of the host and/or the soft and hard tissue metabolism. Up to now, smoking and diabetes are the only risk factors included as periodontitis modifiers in the newly proposed staging scheme [3], while emotional stress and depression are classified as systemic disorders that may contribute to attachment loss [4].

During the past 20 years, the role of psychopathological factors gained attention in periodontitis research. Associations between periodontitis and depression [5], anxiety disorders [6], alcohol consumption and dependence [7], and post-traumatic stress disorder [8] were discussed. However, most studies only investigated the influence of one psychopathology on periodontitis, neglecting important interactions due to psychological comorbidities and social class dependence. These complex interrelations may be one of the reasons why study results are sometimes contradictory. As an example, the association between depression and periodontitis was confirmed in some studies but in others not [9]. For both prevention and therapy of periodontitis, it is of utmost interest to investigate psychosocial factors taking into account their mutual link. The biopsychosocial model of health [10] considers biological, psychological, and social interacting factors as determinants. Within this model, the socioeconomic status plays an important role for either health or disease. Impaired working and living conditions, low education, or low income can provoke pathobiological conditions [11]. Low socioeconomic status can cause chronic stress in an individual, and therefore, biological mechanisms like an activation of the hypothalamic–pituitary–adrenal axis are initiated. Resulting permanent high cortisol levels and a pro-inflammatory immune status can affect the susceptibility to periodontal inflammation [12]. Moreover, dental anxiety is a highly prevalent and important psychological risk factor for oral health impairment [13, 14]. A German survey revealed low levels of dental anxiety in 60%, moderate levels in 23%, and high levels of dental anxiety in 17% of the population studied. Dental phobia was recognized in 11% [15]. Dental anxiety is a common comorbidity of various mental disorders [16]. In own observations, about 42% of patients with post-traumatic stress disorder, 31% with anxiety disorders, and 21% with depressive disorders presented a high degree of dental fear [17]. However, this relationship is seldom recognized and needs further attention within the model of pathogenesis of periodontitis.

Smoking is the only environmental factor that is recognized as true risk factor for periodontitis in the pathogenesis model [2]. It affects the progression rate (higher severity and extent of the disease at an earlier age) as well as the treatment response (less improvement in attachment level and inflammation, higher rates of tooth loss during maintenance care) [3, 18]. DNA damage, a chronic inflammatory state, and oxidative stress provoked by tobacco components are responsible for local and systemic alterations in smokers. Smoking is more prevalent in low social class and in persons suffering from mental illnesses [19, 20]. About twice as many patients with mental disorders smoke compared to the general population [21–23]. At the same time, there is an increased risk in smokers for alcohol-related diagnoses [24], again highlighting the complex interactions between several environmental factors and mental diseases or symptoms.

While still an unclear association between alcohol consumption and periodontitis exists when single studies are considered, a meta-analysis including more than 90,000 participants from 18 studies reported a relative risk for periodontitis of 1.6. A sub-analysis of six studies revealed a linear dose–response relationship between alcohol intake and periodontitis [25]. Only 10 of the 18 single studies included socioeconomic conditions like education and income as covariates. Adjustments for smoking were done in 15 studies. However, comorbidities like depression or dental anxiety were never considered.

The aim of this study was to examine a wide range of psychopathological and socioeconomic factors to identify those primarily associated with periodontitis.

The following hypotheses were studied:

- Periodontitis and non-periodontitis patients differ regarding psychopathological symptom burden.
- Periodontitis and non-periodontitis patients differ regarding socioeconomic status.
- Psychopathological symptoms, social status, and dental parameters are intercorrelated.
- The joint consideration of potential psychosocial risk indicators enables the selection of those factors, which are associated with periodontitis to a relevant extent.

## Methods

In a cross-sectional study, 111 periodontitis patients (periodontitis group) and 110 patients without periodontitis (control group) were consecutively recruited in four urban private dental practices in Germany (Aalen, Berlin, Esslingen, Rottweil). The investigating dentists were trained to apply periodontal examination techniques. The training included oral and written instructions and practice on index assessment and periodontal probing under supervision of a senior clinician. The study took place from January 2015 to February 2016. Written informed consent was obtained from every participant before inclusion. The study was approved by the Ethical Review Board of the Technische Universität Dresden (EK 361092014).

### Participants

Men and women ≥ 40 years of age who gave informed consent and either met criteria for PERIO or NON-PERIO groups were included. All participants received a periodontal examination including probing pocket depths (PD) and clinical attachment loss (CAL). Patients who presented more than 30% teeth with approximal CAL ≥ 5 mm were assigned to the PERIO group [26]. Therefore, all patients were diagnosed with generalized periodontitis stage III or IV as defined in the current classification [3]. Patients who had not more than one tooth with CAL ≥ 4 mm or probing depths ≥ 5 mm qualified for the NON-PERIO group.

Exclusion criteria were:

- Less than 15 teeth
- Severe systemic diseases (e.g., infectious diseases, uncontrolled diabetes)
- Current radiation or chemotherapy
- Prescription of anticoagulants
- Requirement of antibiotic treatment for dental appointments
- Medication intake that affects the gingival condition
- Pregnancy or breastfeeding
- Unability to assess the nature and consequences of study participation

### Parameters

All participants received a dental examination including documentation of decayed, missing, and filled teeth (DMFT). The index represents the sum of diseased teeth in relation to the possible number of teeth (*n* = 28) in a dentition. PD, CAL, bleeding on probing (BOP), plaque index (PI) [27], and gingival index (GI) [28] were documented at six sites per tooth.

In addition, the following questionnaires (all versions in German) to assess psychopathologies, nicotine and alcohol abuse, and socioeconomic burden were used:

- Dental Anxiety Scale (DAS) [29]: four-item questionnaire measuring dental anxiety. Range 4–20. Individuals with a score ≥ 15 were classified as high dental anxious. In addition to the DAS, anxiety-related avoidance behavior was recorded, whereby cancellations/non-attendance of dental appointments and the period of avoiding dental appointments in years were queried.
- Patient Health Questionnaire Depression Scale (PHQ-8) [30]: derived from the depression module of the Patient Health Questionnaire (PHQ-D), the PHQ-8 measures depressiveness with eight items. Range 0–24.
- General Anxiety Disorder (GAD-7) [31] is a 7-item module of the PHQ-D for measuring symptom severity of generalized anxiety. Range 0–21.
- Somatic Symptom Scale (SSS-8) [32] is an 8-item brief version of the PHQ module for accessing perceived burden of common somatic symptoms. Range 0–32.
- Childhood Trauma Screener (CTS) [33] is the short form of the Childhood Trauma Questionnaire for screening recalled abuse and neglect in childhood and youth by five items. Range 5–25.
- Eating disorder (SCOFF) [34] is a screening questionnaire for anorexia nervosa and bulimia nervosa consisting of five dichotomous items. Range 0–5.
- Alcohol Use Disorders Identification Test (AUDIT) [35] is a questionnaire developed on behalf of the WHO to screen for harmful alcohol consumption. With 10 items, alcohol consumption but also drinking behavior and alcohol-related problems are recorded. Range 0–40.
- Fagerström test for nicotine dependence (FTND) [36]: the first item is used to distinguish between smokers, former smokers, and non-smokers. Only current smokers complete six additional questions to capture criteria for nicotine dependence. Based on the total FTND score (range 0 to 10), a categorization into five severity categories can be made. Note, in our regression analysis, we used a modified variable (called FTND*) that includes smoking and currently non-smoking participants (FTND* variable coding: 1 = non-smoker; 2 = former smoker; 3 = severity category, none/very low nicotine dependency (FTND 0–2); 4 = low nicotine dependency (FTND 3–4); 5 = medium nicotine dependency (FTND 5); 6 = strong nicotine dependency (FTND 6–7); 7 = very strong nicotine dependency (FTND 8–10)).
- Socioeconomic Status Index (SES) [31] is a multidimensional index that captures socioeconomic status in the three domains (subscales) income, education, and profession. Score values are assigned to the response categories so that each subscale takes on a quasi-metric value ranging from 1.0 to 7.0 points. The sum of the three scales results in a total score representing socioeconomic status (range 3–21). The higher the sum score, the higher the social status. The scale *education* reflects the individually acquired schooling and professional degree. For the scale *profession* status, the current occupation held by the participant and the main earner in the household is recorded. The *income* scale is derived from household net income weighted by the age-appropriate financial needs of household residents.

The time to answer the questionnaires averaged 30 min. For further information on questionnaire design, validity, and reliability, please refer to the primary literature provided.

### Statistical analysis

Prior to the study, we estimated the minimum sample size required to detect at least medium effect sizes. Medium effects were defined as sufficient, as we aimed to identify differences relevant for clinical practice. To ensure the detection of medium effects (*d* = 0.5) when comparing two equally large, independent groups with a significance level of 5% and a power (1-β) of 0.8, 100 persons per group had to be recruited plus 10% more in case of drop-outs or missing values [37].

Statistical analyses were carried out using SPSS 27 (IBM Corp., Armonk, NY, USA). Missing values were not imputed. Deviations from total *N* within single scales were reported. Medians [$\stackrel{{\sim }}{\text{x}} \,$] and quartiles [*x*_.25_, *x*_.75_] were shown as descriptive values. Since some of the variables were not normal distributed, non-parametric statistical tests were performed. Group differences of categorical variables were examined using Fisher’s exact tests. For ordinal or metric variables Mann–Whitney *U* tests were used, and in addition, Cohen’s *d* was reported as a measure for effect strength. Correlative associations were analyzed using Spearman correlations.

After comparing PERIO and NON-PERIO groups regarding psychological symptom burden and SES, all variables which showed inter-group differences were included in logistic regression analyses (method, forward stepwise likelihood ratio). The regression model was adjusted for age because PERIO patients were significantly older than NON-PERIO patients were.

## Results

### Demographic characteristics

In the PERIO group, 111 participants finished the study. The NON-PERIO group consisted of 110 participants without periodontitis. In both groups, more women were included, but gender distribution did not differ significantly between both groups (Fisher's exact test, *p*=0.056; NON-PERIO female, 65.5% (*n*=72); PERIO female, 52.3% (*n*=58)). Participants in the PERIO group were significantly older then participants from the NON-PERIO group (*U* test, z=4.101; p<0.001; NON-PERIO, $\stackrel{{\sim }}{\text{x}} \,$  = 47.0 years, x_.25_ = 42.0 years,x_.75_ = 56.0 years; PERIO, $\stackrel{{\sim }}{\text{x}} \,$ = 54.0 years,x_.25_=46.0 years, x_.75_ = 63.0 years).

### Clinical parameters

As expected, periodontitis patients had significant higher PD and CAL, more inflammation (BOP, GI) and plaque (PI), and presented less teeth and higher DMFT scores than NON-PERIO group participants (Table 1).

**Table 1:** Group characteristics and group comparisons for dental parameters and socioeconomic and psychopathological factors as well as biopsychosocial associations

Scale	NON-PERIO percentiles			PERIO percentiles			*U* test			Effect size	DMFT		Plaque index[27]		Gingival index[28]		CAL [% teeth > 5 mm]	
	25^th^	50^th^	75^th^	25^th^	50^th^	75^th^	*n*	*z*	*p*	*d*_*Cohen*_	*r*_*S*_	*p*	*r*_*S*_	*p*	*r*_*S*_	*p*	*r*_*S*_	*p*
Dental parameters																		
Number of teeth	24	26	28	21	24	27	221	**4.33**	< 0.001	0.60								
DMFT	8.75	14.00	20.00	14.00	19.00	23.00	221	**4.51**	< 0.001	0.67								
Plaque index [27]	0.19	0.33	0.52	0.70	1.10	1.50	221	**9.46**	< 0.001	1.44								
Gingival index [28]	0.13	0.25	0.62	0.50	0.90	1.41	221	**7.68**	< 0.001	0.84								
BOP [%]	1.79	4.75	8.95	10.42	23.48	46.38	221	**8.74**	< 0.001	1.28								
PD [%teeth PD > 5 mm]]	0.00	0.00	0.00	42.86	60.87	85.00	221	**13.40**	< 0.001	3.38								
CAL [% teeth > 5 mm]	0.00	0.00	0.00	57.69	72.00	95.65	221	**13.42**	< 0.001	5.02								
Socioeconomic status (SES)																		
Status	12.50	16.00	18.70	11.20	13.90	16.70	192	**3.20**	0.001	− 0.49	− 0.110	0.129	** − 0.221**	0.002	** − 0.162**	0.025	** − 0.310**	< 0.001
Education	3.60	6.10	7.00	3.00	3.60	6.10	219	**3.82**	< 0.001	− 0.55	− 0.118	0.081	** − 0.194**	0.004	** − 0.141**	0.036	** − 0.326**	< 0.001
Profession	3.60	4.20	5.20	2.90	3.50	4.20	213	**2.82**	0.005	− 0.37	− 0.062	0.368	** − 0.217**	0.001	** − 0.162**	0.018	** − 0.230**	0.001
Income	5.5	6.5	7.0	4.5	5.5	7.0	198	**2.12**	0.034	− 0.30	− 0.098	0.170	** − 0.188**	0.008	** − 0.153**	0.031	** − 0.229**	0.001
Psychopathologies																		
DAS	5.00	7.00	9.00	7.00	10.00	13.00	221	**5.80**	< 0.001	0.86	**0.255**	< 0.001	**0.286**	< 0.001	**0.334**	< 0.001	**0.373**	< 0.001
GAD-7	0.00	3.00	5.00	1.00	4.00	6.25	211	**2.65**	0.008	0.45	0.100	0.148	0.129	0.061	**0.156**	0.024	0.132	0.055
PHQ-8	0.50	2.00	5.00	2.00	4.00	7.00	210	**2.95**	0.003	0.46	0.074	0.288	**0.184**	0.007	**0.140**	0.043	**0.149**	0.031
CTS	1.00	1.40	1.80	1.20	1.40	1.80	217	**2.51**	0.012	0.34	0.107	0.117	**0.133**	0.050	**0.133**	0.050	**0.174**	0.010
SCOFF	0.00	0.00	1.00	0.00	0.00	1.00	216	0.19	0.852	0.02	0.082	0.228	0.091	0.182	0.058	0.395	− 0.019	0.783
SSS-8	2.00	4.00	6.00	2.00	5.50	10.00	212	**2.55**	0.011	0.42	**0.177**	0.010	**0.191**	0.005	**0.195**	0.004	**0.139**	0.043
AUDIT	3.00	4.00	5.00	2.00	4.00	6.00	190	0.84	0.399	0.15	0.043	0.551	0.134	0.066	0.125	0.087	0.085	0.242
FTND	0.00	1.00	4.00	1.50	4.00	6.00	50	**2.81**	0.005	0.84	0.214	0.136	**0.332**	0.018	0.252	0.077	**0.462**	0.001

In the left part of the table for both groups (patients with periodontitis [PERIO] und without periodontitis [NON-PERIO]) percentiles, group differences (*U* test) and effect sizes (*d*_*Cohen*_) are presented for each scale and dental parameter. The right part of the table presents Spearman’s correlation coefficients [*r*_*S*_] for the associations between dental parameters and socioeconomic or psychopathological variables, respectively. Abbreviations: *DMFT*, number of decayed, missing and filled teeth per dentition; *BOP*, bleeding on probing; *PD*, probing depth; *CAL*, clinical attachment loss; *DAS*, Dental Anxiety Scale; *GAD-7*, General Anxiety Disorder Scale 7; *PHQ-8*, Patient Health Questionnaire 8 Depression Scale; *CTS*, Childhood Trauma Screener; *SCOFF*, Questionnaire to Screen for Eating Disorders; *SSS-8*, Somatic Symptom Scale 8; *AUDIT*, Alcohol Use Disorders Identification Test; *FTND*, Fagerstrom Test For Nicotine Dependence

### Socioeconomic status

Socioeconomic status was significantly lower in the PERIO group compared to the NON-PERIO group (SES *d*_*cohens*_ =  − 0.49). These differences reached statistical significance in all three status domains (income *d*_*cohens*_ =  − 0.30, profession *d*_*cohens*_ =  − 0.37), with the strongest effects for the group differences in the domain education (*d*_*cohens*_ =  − 0.55). SES and its subdomains showed significant negative correlations with the plaque index, gingival index, and CAL. The lower SES, the higher were the index values and attachment loss. However, no significant correlation between SES and DMFT index could be detected (Table 1). Psychopathological symptoms were negatively correlated with SES (all correlations *p* < 0.05; DAS, *r*_s_ =  − 0.233; GAD-7, *r*_s_ =  − 0.147; PHQ-8, *r*_s_ =  − 0.238; CTS, *r*_s_ =  − 0.295; FTND, *r*_s_ =  − 0.304).

### Psychopathological symptom severity

Periodontitis patients reported a higher intensity of psychopathologic symptoms than participants without periodontitis did. The PERIO group reported significant higher dental anxiety (DAS, *d*_*cohens*_ = 0.86), nicotine dependence in smokers (FTND, *d*_*cohens*_ = 0.84), depression (PHQ-8, *d*_*cohens*_ = 0.46), symptoms of general anxiety (GAD-7, *d*_*cohens*_ = 0.45), somatic symptom burden (SSS-8, *d*_*cohens*_ = 0.42), and childhood traumatization (CTS, *d*_*cohens*_ = 0.34) than the NON-PERIO group (Table 1). No statistically significant group differences were found concerning symptoms of eating disorders (SCOFF) or alcohol dependency (AUDIT).

Out of all psychological symptom burden variables, the strongest effect could be observed for dental anxiety. A total of 18.9% (*n* = 21) of patients in the PERIO group suffered from severe dental anxiety compared to only 3.6% (*n* = 4) in the NON-PERIO group. Moderate dental anxiety was found in 11.7% (*n* = 13) of the PERIO participants versus 3.6% (*n* = 4) of NON-PERIO participants, while mild or no anxiety was reported in 69.4% (*n* = 77) PERIO participants compared to 92.7% (*n* = 102) NON-PERIO participants.

Dental anxiety-related avoidance behavior was reported significantly more frequently by PERIO patients. Due to dental fear, 24.5% (*n* = 27) periodontitis patients had already cancelled or have not attended dental appointments compared to 7.3% (*n* = 8) NON-PERIO participants (Fisher's exact test, *n *= 219; *p *< 0.001). Furthermore, 30.9% (*n* = 34) periodontitis patients and 6.4% $(n=7)$ NON-PERIO participants avoided dental appointments for more than 1 year (Fisher's exact test, *n *= 219; *p *< 0.001). Among these patients with anxiety-related avoidance behavior, the median of the longest continuous period of avoiding dental visits was 3 years in the PERIO group ($\stackrel{{\sim }}{\text{x}} \, = 3\mathrm{ years}, {x}_{.25} = 2\mathrm{ years}, {\mathrm{x}}_{.75} = 5\mathrm{ years},\mathrm{ maximum}=18\mathrm{ years}$) and 2 years in the NON-PERIO group (*x* = 2 years, x_.25_ = 1,5 years, x_.75_ = 6 years, maximum = 10 years).

Regarding the number of smokers, the two groups did not differ significantly ($$Fisher^{\prime}s\; exact\,test: n=221; p=0.303$$). Based on patients’ self-reports, 27.9% $$(n=31)$$ of PERIO patients versus 20.9% $$(n=23)$$ of the NON-PERIO participants were current smokers. Furthermore, 37.8% $$(n=42)$$ versus 35.5% $$(n=39)$$ were former smokers and 34% $$(n=38)$$ versus 43.6% $$(n=48)$$ were non-smokers, respectively. However, the groups differed strongly in the severity of nicotine dependence. Periodontitis patients who smoked had a higher nicotine dependence than smokers of the NON-PERIO group ($$FTND, U test:$$ $$n=50; z=2.81; p=0.005;d=0.84)$$.

All variables of psychological symptom burden, which showed significant group differences, significantly correlated with PI, GI, or attachment loss. DMFT index was only correlated with dental anxiety (DAS) and somatic symptom burden (SSS-8) (compare Spearman rank coefficients in Table 1). In addition, intercorrelations existed between psychopathological symptoms. For example, dental anxiety was correlated ($p<0.01$) with symptoms like depressiveness (PHQ-8, ${r}_{S}=0.361; n=210$), general anxiety (GAD-7, ${r}_{S}=0.357;$ *n* = 211), somatic symptoms (SSS-8, ${r}_{S}=0.360;n=212$), and childhood traumatization (CTS, ${r}_{S}=0.164$; *n* = 217).

### Socioeconomic status und psychopathological symptom burden as predictors for periodontitis

A logistic regression analysis was performed to extract variables with the greatest predictive impact on periodontitis (dependent variable, group) out of all potential influencing factors (independent variables, age, SES, DAS, FTND* in smokers, PHQ-8, GAD-7, SSS-8, CTS). Dental anxiety (DAS) could be identified as the strongest predictor in the model. After inclusion of dental anxiety, also age, smoking/nicotine dependence (FTND* in smokers), and socioeconomic status (SES) showed a significant but subordinated influence. All other independent (PHQ-8, GAD-7, SSS-8, CTS) variables had no additional impact and were not included in the model (Table 2).

**Table 2:** Results of regression analyses with group (PERIO vs. NON-PERIO) as dependent variable and age, SES, and relevant psychological symptom burden as independent variables

Variables included in the model		*β*	*p*	OR	95% CI for OR	
Step 1	DAS (*dental anxiety*)	0.233	<0 .001	1.262	1.147	1.389
Step 2	DAS (*dental anxiety*)	0.259	< 0.001	1.295	1.170	1.434
	Age	0.072	<0 .001	1.075	1.036	1.115
Step 3	DAS (*dental anxiety*)	0.247	<0 .001	1.280	1.153	1.420
	FTND* (*smoking*)	0.382	0.008	1.464	1.104	1.942
	Age	0.078	< 0.001	1.081	1.041	1.122
Step 4	DAS (*dental anxiety*)	0.230	< 0.001	1.259	1.133	1.398
	FTND* (*smoking*)	0.348	0.019	1.416	1.060	1.892
	SES (*status*)	−0 .114	0.021	0.892	0.809	0.983
	Age	0.081	< 0.001	1.084	1.042	1.127

Excluded variables: PHQ-8 (*depression*), GAD-7 (*general anxiety*), SSS-8 (*somatic symptom burden*), CTS (*childhood traumatization*)

Standardized regression coefficients β, odds ratios [OR] with 95% confidence interval [CI], and *p* values

## Discussion

There is growing evidence that associations between mental disorders and periodontal health exist. This relationship was supported by our current study results that showed a higher psychopathological symptom burden in periodontitis patients compared to periodontal healthy participants. More specifically, significant group differences were detected concerning depression, general anxiety, dental anxiety, childhood traumatization, and nicotine dependence in smokers.

The relationship between each of the mental disorders and periodontal health has been studied to a varying degree. Study results are contradictory and in parts not consistent and comparable. Concerning depression and periodontitis, one meta-analysis did not support associations between the two conditions and showed high heterogeneity between the studies [5]. Araujo et al. could include only seven cross-sectional studies but criticized statistical, methodological, and clinical heterogeneity. Studies lacked standardized study designs, disease definitions for both periodontitis and depression, and inclusion criteria for participants and outcome variables. Another meta-analysis of 14 studies found an association but explicitly pointed out to interpret these results with care due to a high heterogeneity [38].

The higher rate of self-reported childhood abuse and neglect we found in PERIO patients (*d* = 0.34) has not been investigated so far by the knowledge of the authors. However, this relationship seems obvious, as abuse, especially sexual abuse, predisposes for dental anxiety and avoidance of dental treatment [39, 40]. Our results suggest that the link between childhood trauma and periodontitis may be mediated by dental anxiety, as CTS scores had no significant impact any more when dental anxiety was included in the regression model. The only study that addressed the influence of trauma on periodontal health investigated post-traumatic stress disorders in war veterans [8] and is therefore not comparable in terms of content. In contrast, smoking and nicotine dependency are undisputedly recognized environmental risk factors and modifiers of periodontitis progression [3]. Our results have proven this fact by confirming a higher nicotine dependency in smokers of the periodontitis group.

In addition to the methodological problems critically mentioned by Araujo et al. [5], we also suspect a mis-/over-interpretation of some psychopathological factors due to confounding effects. The investigation of only single psychopathologies ignores the existence of two possible confounders: (i) the comorbidity between mental disorders and (ii) the relation to the socioeconomic status.

(i) There is a high comorbidity between mental diseases [41]. Therefore, psychopathological symptoms cannot be considered as distinct entities. They are more or less correlated with each other. This can be exemplified by the significant correlations of dental anxiety with other symptoms such as childhood traumatization (${r}_{S}=0.164$), depressiveness (${r}_{S}=0.361$), or general anxiety (${r}_{S}=0.357$). It is evident that dental anxiety is often associated with other mental disorders [17, 42]. Patients with depression, anxiety disorder, and post-traumatic stress disorder have a disproportionately high incidence of severe dental fear [17].

(ii) Both mental disorders and periodontitis are more common in the low social class [43, 44]. Our results show that several psychopathologic symptoms and social status are significantly correlated with plaque, gingivitis, and clinical attachment loss. Applying the SES index, comparisons of the influence of the three status domains education, profession, and income can be made. We found the largest group differences in education (*d* = 0.55). Lower education appears to carry a greater risk for periodontitis than lower financial background. Possibly, higher levels of education facilitate access to prevention information and practical understanding of health-promoting behavior. Based on large-scale representative epidemiologic studies, factors like education, income, and poverty–income ratio even when adjusted for age and gender were consistently proven as factors to negatively influence periodontitis [43, 45]. Apparently, low socioeconomic status and periodontitis are interrelated via complex biopsychosocial interactions. Both oral health-related behavioral factors and pro-inflammatory factors like chronic stress [46], metabolic diseases (e.g., diabetes, obesity [47, 48]), and nicotine dependence [18] are relevant risk factors or indicators for periodontitis which are more frequently observed in low social class.

Only by taking comorbidities and socioeconomic status into account, spurious relationships can be uncovered and the complex nature of biopsychosocial interactions understood. The results of the regression model support this statement. As expected, known risk factors like nicotine dependency, age, and social status showed a significant influence on periodontitis. However, the strongest predictor for periodontitis was dental anxiety. Once these factors were included in the model, the other psychopathological symptoms no longer contributed substantially to the prediction of periodontitis. Although dental anxiety and nicotine dependence have a comparably large effect (*d* = 0.86 vs. 0.84), the influence of dental anxiety on periodontitis is still underestimated in research and clinical practice. While associations between caries, dental anxiety, and avoidance behavior were discussed extensively, consequences for periodontal tissues were poorly investigated.

There are a few studies providing evidence for associations between periodontitis and dental anxiety [49–51] and studies which did not confirm associations [52, 53]. However, the validity and comparability of the studies are restricted by methodological limitations and differences. For example, non-validated instruments or single-items were used to assess dental anxiety [51, 52], periodontitis was documented based on self-reports instead of clinical examinations [50], participants were recruited selectively (e.g., only male soldiers) [53], and the studies differed widely in statistical power and thus in detectable effect sizes.

Similar to our approach, Guentsch and co-workers [49] recruited participants in a German dental practice and assessed dental anxiety using DAS and periodontal status by clinical examination. In accordance with our results, a negative effect of dental anxiety on dental and periodontal health was confirmed. In their study, highly anxious patients (MDAS ≥ 19) compared to lower anxious patients (MDAS < 19) reported more often infrequent dental treatment and had more decayed and fewer filled teeth and more BOP indicating a higher degree of inflammation. Although their group comparison did not reach the significance level for attachment loss, this should not be interpreted per se as a negative result or contradiction to our findings. It might be due to the small number of cases in Guentsch’s highly anxious group (*n* = 20). Their reported group means and standard deviations corresponded to a medium effect and suggested a trend towards greater attachment loss in high dental anxious patients.

Considering methodological limitations discussed above, two large-scale Chinese studies strengthen the evidence for dental anxiety associated impairments of periodontal health. A representative cross-sectional study in Hong Kong (*N* = 1000) showed significantly higher CAL in participants with self-reported higher dental anxiety versus low or non-anxious participants [51]. Liu and co-workers [50] found significantly higher dental anxiety (using DAS and DFS) in participants with self-reported periodontal disease compared with those without periodontitis. In a recent preprint report, Liu and co-workers even point to a dental anxiety-reducing effect of periodontitis therapy with scaling and root planing [54]. Of even greater importance would be the evaluation of long-term efficacy of anxiety treatment for periodontal health. Future studies on psychotherapy for dental anxiety should include dental and periodontal parameters as secondary outcomes, in addition to anxiety reduction as the main efficiency criterion. As dental anxiety responds very well and quickly to cognitive-behavioral therapy [55], it could be an easily modifiable factor to improve dental care utilization. Even one-session treatments and large group exposure treatment can reduce dental anxiety significantly [56, 57].

For clinical implications, this knowledge of psychosocial risk indicators of periodontitis can sensitize dentists to pay attention to information deficits or fear-related avoidance of prevention and treatment services in socially disadvantaged and dentally anxious patients. Based upon this knowledge, dentists can support affected patients individually. However, dentists cannot reach patients who avoid visiting the dentist. Therefore, it is of particular importance that physicians and psychotherapists are informed about periodontitis and its risk indicators. They can become aware of dental anxiety, oral diseases, and avoidance of regular dental visits and contribute to early detection and referral to specialist care.

### Strengths and limitations of the method

As a cross-sectional survey, our study does not permit causal interpretations. The sample represented the typical clientele of patients in German standard dental practices but was not representative for the population. Our recruitment design entailed a selection bias. It excluded individuals who totally avoid dental visits from participating in the study. Patients with dental phobia are underrepresented in dental offices. Therefore, we possibly even underestimate the influence of dental phobia on periodontitis.

Psychopathological symptom severity was assessed using validated and internationally established questionnaires. As short and easy-to-interpret instruments, they are also practical for clinical routine and frequently used as screening instruments. However, no diagnosis should be made on the basis of questionnaires alone. In the case of retrospective self-reports such as childhood traumatization, the possibility of memory bias must be noted [58].

With the SES index, a validated instrument was used that captures socioeconomic status in a very detailed fashion. It enables to compare and to quantify the status domains and provides an easily manageable variable for statistical analyses due to its metric total score [59]. Nowadays, such multidimensional status indices are preferred in large epidemiological studies. For further periodontitis research, it would be desirable to increase the use of sound methods for capturing the complex construct of social status. Instead of the often self-constructed items and response categories, we recommend the use of validated instruments that allow comparability between studies.

## Conclusions

Out of a broad spectrum of psychopathological symptoms, dental anxiety and low socioeconomic status, especially low education, are associated with periodontitis.
